# Splenic Rupture Secondary to Vascular Ehlers–Danlos Syndrome Managed by Coil Embolization of the Splenic Artery

**DOI:** 10.1055/s-0039-3399555

**Published:** 2019-11-22

**Authors:** Keisha C. Kamalanathan, Alex M. Barnacle, Charlotte Holbrook, Clare Rees

**Affiliations:** 1 Department of Interventional Radiology, Great Ormond Street Hospital for Children NHS Foundation Trust, London, United Kingdom of Great Britain and Northern Ireland; 2 Department of Neonatal and Paediatric Surgery, Great Ormond Street Hospital for Children NHS Foundation Trust, London, United Kingdom of Great Britain and Northern Ireland

**Keywords:** splenic rupture, embolization, Ehlers–Danlos syndrome, vascular EDS, pediatric

## Abstract

**Aim** Atraumatic splenic rupture is uncommon and life-threatening. It may be related to underlying pathology and be the initial manifestation of the condition. Vascular Ehlers–Danlos syndrome (V-EDS) is a rare autosomal dominant collagen vascular disorder, associated with vessel fragility and rupture. We describe a child presenting with splenic rupture managed by embolization of the splenic artery. She was subsequently diagnosed with V-EDS.

**Case Description** A 11-year-old girl with thalassemia trait presented with sudden onset of abdominal pain and hypovolemic shock. There was no history of trauma. Following resuscitation, abdominal computed tomography showed hemoperitoneum and active splenic arterial extravasation. Angiography demonstrated four bleeding points, from irregular vessels supplying the upper two-thirds of the spleen. These were not amenable to supraselective embolization. Therefore, coil embolization of the main splenic artery was performed, with no splenic supply seen on the postembolization angiogram. Her postoperative recovery was complicated by pancreatitis secondary to partial ischemia of the pancreatic tail. Subsequent extensive investigations excluded hematological, myeloproliferative, and infective causes for her splenic rupture. A safeguarding investigation was completed, with no pertinent factors identified. Findings of thin skin, abnormal bruising, and hypermobile joints raised a clinical suspicion of a connective tissue disorder. Genetic testing revealed a de novo mutation of the COL3A1 gene.

**Conclusions** There are only four reports of V-EDS causing splenic rupture in the literature to date. These patients were all adults and only one had not previously been diagnosed with V-EDS. All underwent splenectomy. While V-EDS presenting with abdominal visceral rupture in children has been reported, this is the first report of a child with V-EDS presenting with splenic rupture. It is the only case of splenic rupture secondary to V-EDS that has been managed minimally invasively by embolization.

## Introduction

Splenic rupture, if not recognized and managed appropriately, is a potentially life-threatening event. In children, it most commonly occurs secondary to trauma and is often managed conservatively. Spontaneous splenic rupture is a rare occurrence and is usually a consequence of an infective or infiltrative process. The Ehlers–Danlos syndromes (EDS) are a group of rare inherited conditions that affect connective tissue. These disorders are characterized by tissue fragility of the skin, ligaments, blood vessels, and internal organs. Vascular EDS (V-EDS) is the rarest form of EDS and carries the highest mortality rate, due to its association with dissection of major blood vessels or rupture of solid organs. 1

## Case Report

**Fig. 1:**
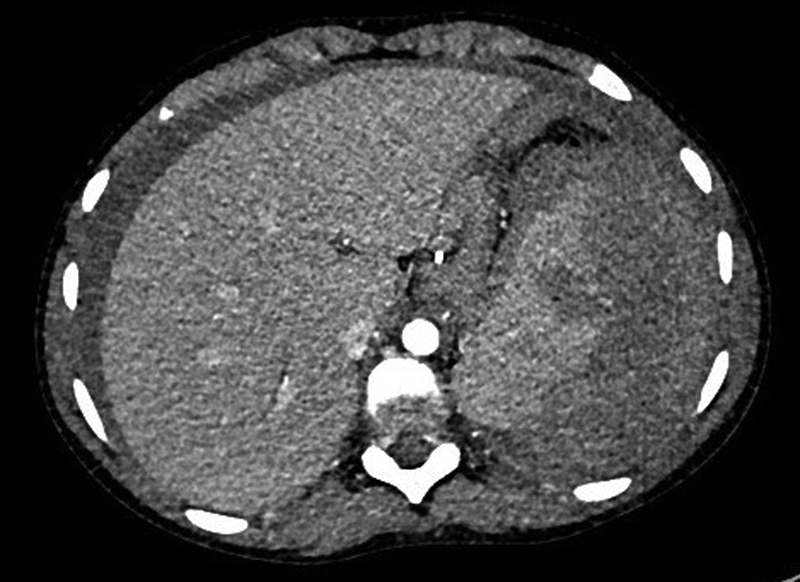
Axial image from the contrast-enhanced computed tomography scan performed on admission, showing rupture of the lateral margin of the spleen and a large perisplenic hematoma. Free fluid is also seen lateral to the liver.

**Fig. 2:**
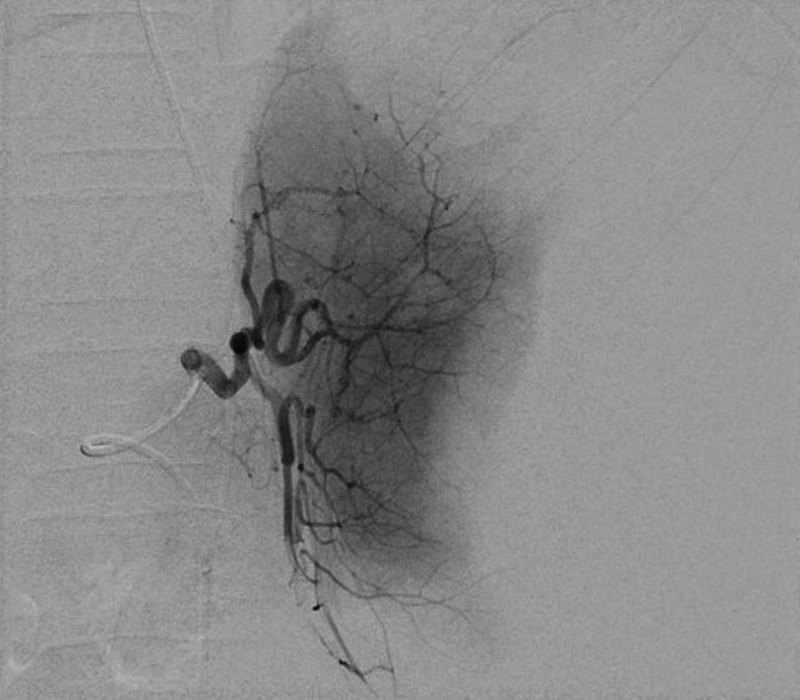
Digitally subtracted image from the splenic artery angiogram, showing distortion of the splenic anatomy by the hematoma, loss of parenchymal perfusion in the lateral part of the spleen and multiple areas of vessel irregularity, and contrast extravasation in the upper pole of the spleen.

A 11-year-old girl presented to her local hospital with an acute onset of severe abdominal pain, showing signs of hypovolemic shock and requiring emergency resuscitation prior to transfer to a tertiary pediatric center. She had fainted at school earlier in the day and been sent home. That afternoon, she had vomited once and rested in bed. She woke during the night, screaming in pain. An ultrasound scan at the local hospital demonstrated a ruptured spleen with an associated perisplenic hematoma and a large volume of free fluid in the peritoneal cavity. These findings were confirmed on a contrast-enhanced computed tomography scan ( Fig. 1 ), which also demonstrated active extravasation of contrast from a branch of the splenic artery. After further resuscitation at the tertiary center, including multiple blood product transfusions, she was transferred to the interventional radiology suite where angiography via a femoral arterial approach showed gross disorganization of the splenic vasculature at the site of parenchymal rupture, with several truncated vessels and active bleed points noted ( Fig. 2 ). Distal embolization of individual bleeding vessels proved impossible due to anatomical distortion by the surrounding hematoma and vessel spasm. In view of the emergency nature of the situation, coil embolization of the proximal splenic artery was performed, with complete occlusion of splenic arterial flow. This inevitably involved partial devascularization of the pancreatic tail.

The patient's vital signs and transfusion requirements improved immediately after coil deployment. She made a slow but steady recovery on the intensive care unit and was extubated 3 days after admission. Her recovery was complicated by transient pancreatitis that was conservatively managed. She was discharged from the hospital 4 weeks later. Follow-up showed reconstitution of most of the splenic parenchyma, though she was vaccinated and commenced on prophylactic antibiotics in view of presumed hyposplenism.

The child's clinical presentation gave no clues as the cause of her splenic rupture. Among other investigations, a referral was made to the children's social care services to explore the possibility of unreported trauma or abuse in view of the lack of any medical explanation for her injury; these enquiries revealed nothing of concern. She was born in the United Kingdom, though her family was from northern Iran; her parents were consanguineous. She was the oldest child of four siblings and had no family medical history of interest. She had been investigated at her local hospital 2 years previously for faltering growth; blood tests had revealed minor anemia, β thalassemia trait, and a spleen at the upper limit of normal for size, but all other tests were normal. She was underweight and was noted to bruise and scar easily. She had no history of fractures or dislocations and no joint pain. On examination, she had thin skin that was slightly hyperextensible and had multiple scars over her knees and elbows. She had blue sclerae. She had no signs of significant hypermobility.

Subsequent genetics investigations confirmed that she was heterozygous for the de novo c.2194G > A mutation in the COL3A1 gene, in keeping with nonhereditary V-EDS. Imaging investigations showed mild fusiform dilatation of the origin of her superior mesenteric artery but otherwise normal vasculature. Cardiac, rheumatological, and surgical follow-up has been unremarkable. She is maintained on regular β-blockers as a protective measure against arterial dissection/rupture and avoids contact sports.

## Discussion

Splenic rupture secondary to V-EDS has been reported rarely in the literature but never in a young child. 2 Vascular, or type 4, EDS is an autosomal dominant connective tissue disorder caused by a mutation in type III procollagen (COL3A1) and carries the highest mortality rate of the EDS subtypes. 1 3 4 5 It is associated with translucent skin, easy bruising, and an increased risk of arterial, intestinal, and solid organ rupture. It is unusual to find joint hypermobility and skin hyperextensibility that is characteristic of the classical EDS, in V-EDS. 1 Fatal complications are reported in up to 70 to 80% of affected individuals before the age of 40. 1 3 Therefore, establishing an early diagnosis of V-EDS is of utmost importance. 3 5 Regular cardiac review and imaging surveillance are required and patients should be counseled about the risks of contact sports, pregnancy, and childbirth. 5 Surgical outcomes in this patient group can be poor due to the underlying fragility of the connective tissues; percutaneous interventions such as embolization carry the advantage of minimal tissue trauma and can be life-saving in a clinical emergency. 6 Nevertheless, the procedure is not without its complications. Reported complications following splenic embolization in the emergency setting (typically after blunt trauma, rather than spontaneous rupture) include rebleeding, infarction, and infection/abscess. 7 Pancreatitis following proximal splenic artery embolization is not commonly reported, but transient elevation of pancreatic enzymes was reported in 36% of cases in a series of 12 adults undergoing embolization for splenic artery aneurysms and false aneurysms. 8 Pancreatitis has been reported after transcatheter embolization of a splenic aneurysm 9 and several case reports of severe necrotizing pancreatitis following splenic artery embolization after trauma. 10 11 12 Therefore, although pancreatitis may be an uncommon complication after this procedure, it should be taken into consideration, due to its potential to result in significant morbidity.

## Notes

New Insights & the Importance for the Pediatric Surgeon Vascular Ehlers–Danlos syndrome is an exceedingly rare but recognized cause of life-threatening spontaneous splenic rupture. Splenic hemorrhage can be swiftly and effectively managed by percutaneous arterial embolization.
